# Prevalence of Trachoma in the North Region of Cameroon: Results of a Survey in 15 Health Districts

**DOI:** 10.1371/journal.pntd.0002932

**Published:** 2014-06-12

**Authors:** Blaise Noa Noatina, Giles Kagmeni, Yaya Souleymanou, Henri Claude Moungui, Ann Tarini Hien, Julie Akame, Yaobi Zhang, Assumpta Lucienne Françoise Bella

**Affiliations:** 1 Programme National de Lutte Contre la Cécité, Ministère de la Santé, Yaoundé, Cameroun; 2 Faculté de Médecine et des Sciences Biomédicales, Université de Yaoundé I, Yaoundé, Cameroun; 3 Délégation Régionale de la Santé Publique du Nord, Garoua, Cameroun; 4 Délégation Régionale de la Santé Publique de l'Extrême-Nord, Maroua, Cameroun; 5 Helen Keller International, Yaoundé, Cameroun; 6 Helen Keller International, Regional Office for Africa, Dakar, Senegal; 7 Faculté de Médecine et des Sciences Biomédicales, Université de Yaoundé I, Yaoundé, Cameroun; University of Cambridge, United Kingdom

## Abstract

**Background:**

To estimate the prevalence of trachoma in the North Region of Cameroon in order to facilitate the planning of trachoma control activities in this region, a survey was carried out in 2011 and 2012 in 15 health districts (HDs).

**Methodology:**

A cross-sectional, two-stage cluster random sampling survey was carried out. The survey focused on two target populations: children aged 1 to 9 years for the prevalence of Trachomatous Inflammation-Follicular (TF) and those aged 15 and over for the prevalence of Trachomatous Trichiasis (TT). The sample frame was an exhaustive list of villages and neighborhoods of HDs. The World Health Organization simplified trachoma grading system was used for the recognition and registration of cases of trachoma.

**Principal Findings:**

30,562 children aged 1 to 9 years and 24,864 people aged 15 and above were examined. In children aged 1–9 years, the overall prevalence of TF was 4.2% (95% confidence intervals (CI): 4.0–4.5%). Three (3) of 15 HDs in the region showed TF prevalence of ≥10% (Poli, Rey Bouba, and Tcholliré). The overall TT prevalence was 0.25% (95% CI: 0.20–0.33%). There were estimated 1265 TT cases in the region. The prevalence of blindness was 0.01% (95% CI: 0.00–0.03%), low vision was 0.11% (95% CI: 0.07–0.17%), and corneal opacity was 0.22% (95% CI: 0.17–0.29%).

**Conclusions/Significance:**

This survey provides baseline data for the planning of activities to control trachoma in the region. The overall prevalence of TF in the region is 4.2%, and that of TT is 0.2%; three HDs have a TF prevalence ≥10%. These three HDs are eligible for mass drug administration with azythromycin, along with the implementation of the “F” and “E” components of the SAFE strategy.

## Author Summary

Trachoma is the most common infectious cause of blindness. Recurrent episodes of infection with *Chlamydia trachomatis* cause conjunctival inflammation in children who go on to develop scarring and blindness as adults. The current effort led by the World Health Organization (WHO) and the Alliance for Global Elimination of Blinding Trachoma by the year 2020 (GET2020) is to eliminate trachoma as a blinding disease worldwide by the year 2020 using the SAFE strategy (Surgery, Antibiotics, Facial cleanliness and Environmental improvement). To estimate the prevalence of trachoma in the North Region of Cameroon in order to facilitate the planning of trachoma control activities in this region, a survey was carried out in 2011 and 2012 in 15 health districts (HDs). The overall prevalence of TF in the region is 4.2%, and that of TT is 0.25%; three HDs have a TF prevalence ≥10%. These three HDs are eligible for mass drug administration with azythromycin, along with the implementation of the “F” and “E” components of the SAFE strategy. There were estimated 1265 TT. This survey provides baseline data for the planning of activities to control trachoma in the region.

## Introduction

Trachoma is the commonest infectious cause of blindness. Recurrent episodes of infection with *Chlamydia trachomatis* cause conjunctival inflammation in children some of whom may go on to develop scarring and potentially become blind as adults. Trachoma affects about 21.4 million people of whom about 2.2 million are visually impaired and 1.2 million are blind [1]. It was once endemic in most countries. It is responsible, at present, for more than 3% of the world's blindness but the number keeps changing due to the effect of socioeconomic development and current control programmes for this disease.

The disease is largely found in poor, rural communities in developing countries, particularly in sub-Saharan Africa [2]. The current effort led by the World Health Organization (WHO) and the Alliance for Global Elimination of Blinding Trachoma by the year 2020 (GET2020) is to eliminate trachoma as a blinding disease worldwide by the year 2020 using the SAFE strategy (Surgery, Antibiotics, Facial cleanliness and Environmental improvement) [3], [4], [5], [6].

Before activities are planned in a given region, data need to be collected on the epidemiological characteristics of active and cicatricial (late stage) disease. In Cameroon, two previous surveys have showed that trachoma is a public health issue in the Far North Region [7], [8]; the North Region share common feature of poverty, dryness, dusty environment and poor sanitation with the Far North Region [9].

The North Region is divided into 15 Health Districts (HDs), 145 Health Areas. Health facilities include: 1 Regional Hospital, 15 District Hospitals, 191 Integrated Health Centres, 6 subdivisional medical centres, and 8 private clinics and medical offices. In the framework of the current primary health care (PHC) structure, the health centres act as the interface between the communities and the health services, dispensing integrated, continuous and comprehensive health care. District hospitals are secondary care institutions and are the referral points for all health centres in the districts. The district health team manages and supervises health centres as well as the district hospital, and is responsible for planning and coordination of services, training and information management and analysis, monitoring and evaluation of program implementation. The regional health team assures the coordination, training, supervision, supplies, monitoring and evaluation of the health districts within the region. Communities also participate in service set up, management, short term and long term planning, financing, monitoring and evaluation.

To assess the epidemiological situation of trachoma in the North Region of Cameroon, an epidemiological survey was carried out in the 15 HDs of the North Region in 2011 and 2012. Prevalence of trachomatous inflammation – follicular (TF) in children aged 1 to 9 years and prevalence of trachomatous trichiasis (TT) in people aged 15 years and over were evaluated. This paper presents the distribution of trachoma in the region and discusses the interventions needed to eliminate the blinding disease from the region.

## Methods

### Ethics statement

The survey was part of the national trachoma control program and was carried out by the National Blindness Control Program of the Ministry of Public Health according to the WHO endorsed survey methodology [10]. Prior to the survey, an ethical clearance was obtained from National Ethics Committee and an administrative authorization was obtained from the Ministry of Public Health. The Health District survey team visited villages, gave information about trachoma (causes, transmission, signs of the disease, how to prevent and treat), and explained the purpose of the survey. Verbal consent for the participation was sought from each participant aged 15 and above and for children aged 1 to 9 years consent was systematically sought from the family heads or guardians. Verbal consent was obtained because most people surveyed were illiterate. The National Ethics Committee and the Ministry of Public Health approved the use of oral consent. After ensuring that the potential subject has understood the information, the enumerator signed and dated the consent form and wrote his name and the family head's name on the form. The village head witnessed the all process. A copy of information notice was handed to those who could read French.

### Survey settings

The North Region makes up 66,090 km^2^ of the northern half of The Republic of Cameroon. It borders the Adamawa Region to the south, Chad and Central African Republic to the east, Nigeria to the west and the Far North Region to the north. The region is hot and dry with seasonal waterways. All rivers in the region experience a tropical regime with a period of high water during the rainy season during which flooding may occur. The rivers mostly dry up during the dry season, many disappearing completely into the sand. From the Bénoué Depression south, the North Region experiences tropical climate of the Sudan type. North of the Bénoué Depression, a Sahel climate prevails. The rainfall varies locally from 500 to 1500 mm per year. The population of the region was estimated at 2 050 229 inhabitants in 2010 [11]. The population density is 31 inhabitants/Km^2^, which means in the Cameroonian context, the North Region is a moderately populated region. 72.1% of the population lives in the rural area and 48.5% is less than 15 years [11]. Major ethnic groups include the Fulani, who are Islamic pastoralists, and numerous Muslim and animist speakers of Adamawa, Chadic, and Nilo-Saharan languages. The region is divided into 15 HDs. The survey was done mainly in 2011 in 14 HDs and in 2012 in one HD (Garoua 1).

### Survey design and sampling

The survey methodology used in this survey was the same as used in the previous survey in the Far North Region and hence the text in the Methods section here, described below, largely overlaps with the previous paper [8]. It was a cross-sectional two-stage random cluster sampling design according to the WHO recommendations [10]. For TF prevalence survey, the sample size for children aged 1–9 years for each district was calculated using an expected TF prevalence of 20% based on Kolofata survey data from the Far North Region, a desired precision of estimate of 3.5%, an alpha risk of 5%, and a design effect of 4. An extra 5% was added to adjust for non-respondents and other recording errors. This resulted in a sample of 2107 children aged 1–9 years per district, approximately 70 children per cluster.

For TT prevalence survey in persons aged 15 and above, the sample size was calculated using an expected TT prevalence of 2.5%, a desired precision of estimate of 1%, an alpha risk of 5%, and a design effect of 1.5. An extra 5% was also added to the sample size to adjust for non-respondents and other recording errors, giving rise to a sample size of 1475 per district, approximately 50 persons each cluster.

Within each HD, 30 clusters (villages) were randomly selected at the first stage on the basis of probability proportional to population size and cumulative totals from a list of villages in the district. Then, selection of households was made within the clusters using the compact segment sampling method [12]. The villages were divided into segments that contained approximately the same number of households. Segments were then randomly selected until enough numbers of children aged 1 to 9 years and those aged 15 and above were reached.

### Household survey

Within each household, all eligible household members (who have resided at least six months in the village or neighborhood at the time of survey) including those absent at the time of visit were identified and enumerated for examination. Those household members present at the time of survey and willing to participate in the survey were examined.

The WHO simplified trachoma grading system was used for the recognition and registration of cases of trachoma [13], [14]. Ophthalmic nurses or ophthalmic medical assistants, some of whom participated in the Kolofata survey in 2006 and in the Far North region survey in 2010–2011, were selected for training as trachoma graders. They were trained for 4 days. The first three days were classroom training on standardized procedures for selection of households, enumeration, examination, identification of trachoma, and collecting and managing data. For the recognition of signs of trachoma, slides showing pictures of various forms of trachoma were used. They were trained on reliably recognizing TF, TT and corneal opacity (CO) signs using these pictures and on safely examining eyes of adults, children and infants. The training also included discussions on how to prevent transmission of eye infections from one person to another during the process of examination and on how to recognize the clinical signs of other national priority eye diseases (i.e. cataract, glaucoma, and refractive errors) and how to refer or treat such patients. At the end of classroom training, the trainees were projected 100 pictures showing various signs of trachoma and were tested in their ability to correctly identify the trachoma grades on an answer paper. Those who had 90% of correct answers were finally recruited as trachoma graders in the team. The training sessions in the field on the fourth day included household selection and enumeration only.

The eyelids and cornea were first examined for deviated lashes and/or corneal opacities. When such signs were found, visual acuity was measured using Snellen E optotypes. If there was visual loss, trachoma accountability was sought (presence of conjunctival trachomatous scarring (TS), TT and/or pannus). The upper eyelids were routinely everted and examined using a binocular loupe (magnification 2.5), under good light conditions. Those with blindness or visual impairment were reviewed by a senior ophthalmologist to make sure the situation was imputable to trachoma. The diagnosis of both eyes was recorded on the data collection form. The surveyors (trachoma graders) ensured that the data collection form was completed in accordance to protocol requirements before proceeding to the next person. In cases that different grades of trachoma were observed between both eyes, the more serious condition was used for the individual. The definitions for visual impairment, low vision and blindness used in the present study followed those given in the Section H54 of the International statistical classification of diseases and related health problem, 10th revision (ICD-10) [15]. The details include:

- Visual impairment includes low vision as well as blindness;
- Low vision is defined as visual acuity of less than 6/18, but equal to or better than 3/60, or a corresponding visual field loss to less than 20 degrees in the better eye with best possible correction (ICD-10 visual impairment categories 1 and 2);
- Blindness is defined as visual acuity of less than 3/60, or a corresponding visual field loss to less than 10 degrees in the better eye with best possible correction (ICD-10 visual impairment categories 3, 4 and 5).

All those who showed signs of active trachoma were treated locally with 1% tetracycline ointment, twice daily for 6 weeks. TT cases were referred to an ophthalmologic center for free surgical intervention.

### Data management and analysis

Data were entered into database using the EpiInfo software for analysis. Data were cleaned, and entries missing or invalid data, particularly on age, were not included in the final analysis. When calculating the overall prevalence in the region or prevalence by sex, samples were weighted according to the proportion of population in each district among the total population in 2010 in the region projected from the 2005 national census with an annual growth rate of 2.6% [11]. The 95% confidence intervals (CIs) of the prevalence were calculated with the Wilson score method without continuity correction [16], using the CI calculator (available: http://vl.academicdirect.org/applied_statistics/binomial_distribution/ref/CIcalculator.xls). Differences in prevalence of trachoma were compared using the Chi-squared test in EpiInfo (version 7.1.1.14). Geographical prevalence maps were drawn using ArcGIS version 10 (ESRI, Inc). Trichiasis cases in each district were estimated according to the population in 2010 and the overall TT prevalence in each district.

## Results

### Study population

A total of 30,881 children aged 1–9 years were enumerated from the selected households, and 30,562 were examined in the 15 HDs surveyed. This represents a participation rate of 99.0%. A total of 25,095 people aged 15 and above were identified and 24,864 were examined, representing a participation rate of 99.1%. Table 1 shows the demographic data of the surveyed population in each district. In total, 55,976 persons were identified and 55,426 were examined, an overall participation rate of 99.0%. The females represented 55.7% in the total number of people aged 15 years and above examined and 48.5% in the total number of children aged 1–9 years examined.

**Table 1:** Demographic data of the surveyed population in each health district.

District	Estimated population in 2010	No of clusters surveyed	No of children (1–9 years) enumerated	No of children (1–9 years) examined	Participation rate (%)	Proportion of females (%)	No of adults (≥15 years enumerated)	No of adults (≥15 years) examined	Participation rate (%)	Proportion of females (%)
BIBEMI	124 535	30	2202	2202	100.0	49.5	1729	1729	100.0	55.3
FIGUIL	91 745	30	2179	2179	100.0	48.6	2150	2150	100.0	59.3
GAROUA 1	229 870	30	1906	1691	88.7	44.7	1421	1272	89.5	53.1
GAROUA 2	229 406	30	2126	2126	100.0	48.8	1589	1589	100.0	59.2
GASCHIGA	94 133	30	1764	1756	99.5	49.7	1915	1883	98.3	52.3
GOLOMBE	67 855	30	2004	2004	100.0	44.9	1526	1526	100.0	44.7
GUIDER	224 726	30	1798	1798	100.0	51.6	1428	1428	100.0	61.5
LAGDO	141 889	30	2115	2115	100.0	49.6	1544	1544	100.0	59.6
MAYO OULO	128 556	30	2562	2557	99.8	48.8	2017	2017	100.0	65.6
NGONG	181 408	30	2005	2005	100.0	49.9	1484	1484	100.0	50.4
PITOA	152 544	30	2178	2178	100.0	48.5	1652	1649	99.8	53.8
POLI	77 024	30	2090	2077	99.4	48.1	2014	1979	98.3	55.9
REY BOUBA	97 881	30	2161	2151	99.5	49.5	1622	1619	99.8	60.2
TCHOLLIRE	128 076	30	1678	1678	100.0	48.0	1424	1424	100.0	49.9
TOUBORO	165 722	30	2113	2045	96.8	47.4	1580	1571	99.4	50.5
**TOTAL**	**2 135 370**		**30881**	**30562**	**99.0**	**48.5**	**25095**	**24864**	**99.1**	**55.7**

### Prevalence of TF in children aged 1–9 years

**Figure 1:**
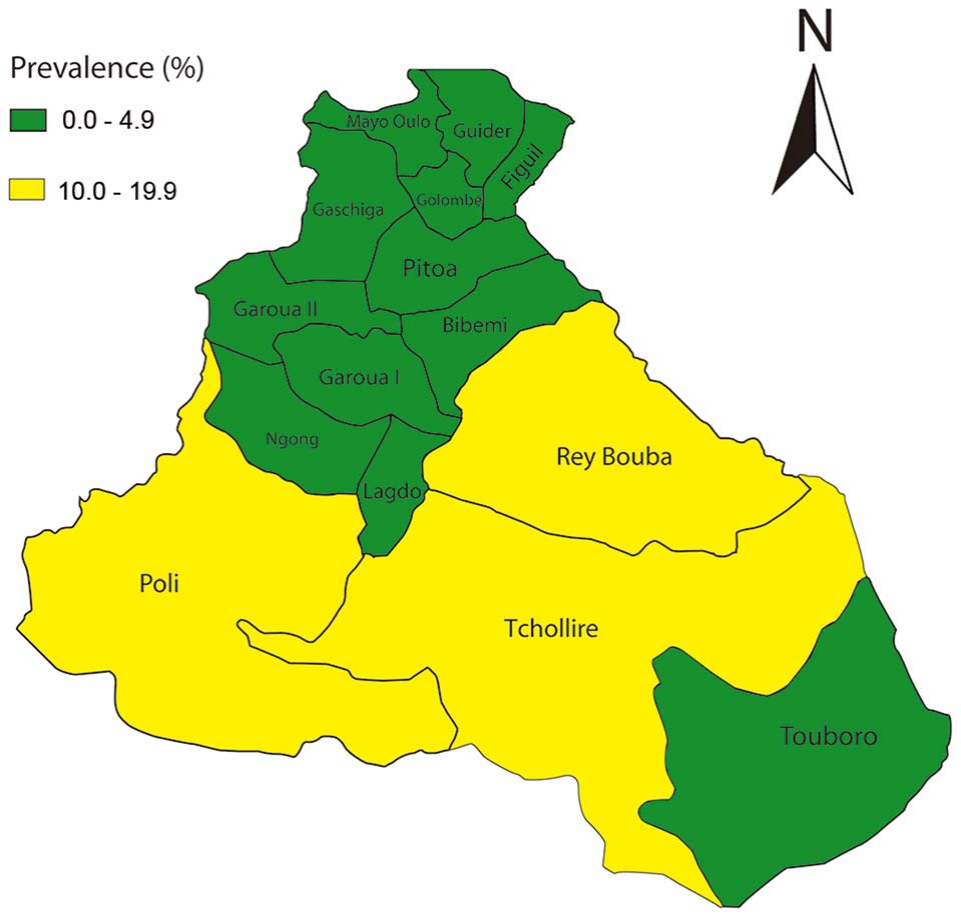
TF prevalence in the North Region Cameroon.

Table 2 summarizes the prevalence of trachoma in children aged 1–9 years in the North Region by district. The overall TF prevalence was 4.2% (95% CI: 4.0–4.5%) in the region, ranging from 0% to 14.5% among 15 districts surveyed. There were three (3) HDs showing TF prevalence of ≥10% (Figure 1). These were Poli, Rey Bouba, and Tcholliré HDs.

**Table 2:** Prevalence (%) of trachoma and estimated TT cases in the North Region.

District	No of children (1–9 years) examined	TF in children aged 1–9 years (95% CI)	Active trachoma (TF&TI) in children aged 1–9 years (95% CI)	No of adults (≥15 years) examined	TT in adults ≥15 years (95% CI)	TT in all ages (95% CI)	Estimated TT cases
BIBEMI	2202	3.6 (2.9–4.4)	3.6 (2.9–4.4)	1729	0.1 (0.0–0.3)	0.1 (0.0–0.2)	70
FIGUIL	2179	1.9 (1.4–2.6)	1.9 (1.4–2.6)	2150	0.0 (0.0–0.0)	0.0 (0.0–0.0)	0
GAROUA 1	1691	0.4 (0.2–0.8)	0.4 (0.2–0.8)	1272	0.3 (0.1–0.8)	0.2 (0.1–0.4)	259
GAROUA 2	2126	3.9 (3.2–4.8)	4.0 (3.3–5.0)	1589	0.0 (0.0–0.0)	0.0 (0.0–0.0)	0
GASCHIGA	1756	3.6 (2.8–4.6)	3.6 (2.8–4.6)	1883	0.0 (0.0–0.0)	0.0 (0.0–0.0)	0
GOLOMBE	2004	0.0 (0.0–0.0)	0.0 (0.0–0.0)	1526	0.0 (0.0–0.0)	0.0 (0.0–0.0)	0
GUIDER	1798	4.6 (3.7–5.6)	7.2 (6.1–8.5)	1428	0.0 (0.0–0.0)	0.0 (0.0–0.0)	0
LAGDO	2115	1.7 (1.2–2.3)	1.7 (1.2–2.3)	1544	0.0 (0.0–0.0)	0.0 (0.0–0.0)	0
MAYO OULO	2557	0.0 (0.0–0.0)	0.0 (0.0–0.0)	2017	0.0 (0.0–0.0)	0.0 (0.0–0.0)	0
NGONG	2005	0.5 (0.3–1.0)	0.7 (0.4–1.2)	1484	0.1 (0.0–0.4)	0.0 (0.0–0.2)	0
PITOA	2178	4.3 (3.5–5.2)	5.4 (4.5–6.4)	1649	0.1 (0.0–0.4)	0.1 (0.0–0.2)	86
POLI	2077	12.6 (11.3–14.1)	13.7 (12.3–15.2)	1979	1.8 (1.3–2.4)	0.9 (0.7–1.3)	391
REY BOUBA	2151	10.6 (9.4–12.0)	11.6 (10.3–13.0)	1619	0.2 (0.1–0.5)	0.1 (0.0–0.3)	55
TCHOLLIRE	1678	14.5 (12.9–16.3)	14.7 (13.9–16.5)	1424	0.4 (0.2–0.9)	0.3 (0.2–0.6)	217
TOUBORO	2045	3.0 (2.4–3.9)	3.1 (2.4–3.9)	1571	0.4 (0.2–0.9)	0.2 (0.1–0.4)	187
**TOTAL**	**30562**	**4.2 (4.0–4.5)**	**4.6 (4.4–4.9)**	**24864**	**0.2 (0.2–0.3)**	**0.1 (0.1–0.2)**	1265

### Prevalence of TT, low vision and blindness due to trachoma in people aged 15 and above

**Figure 2:**
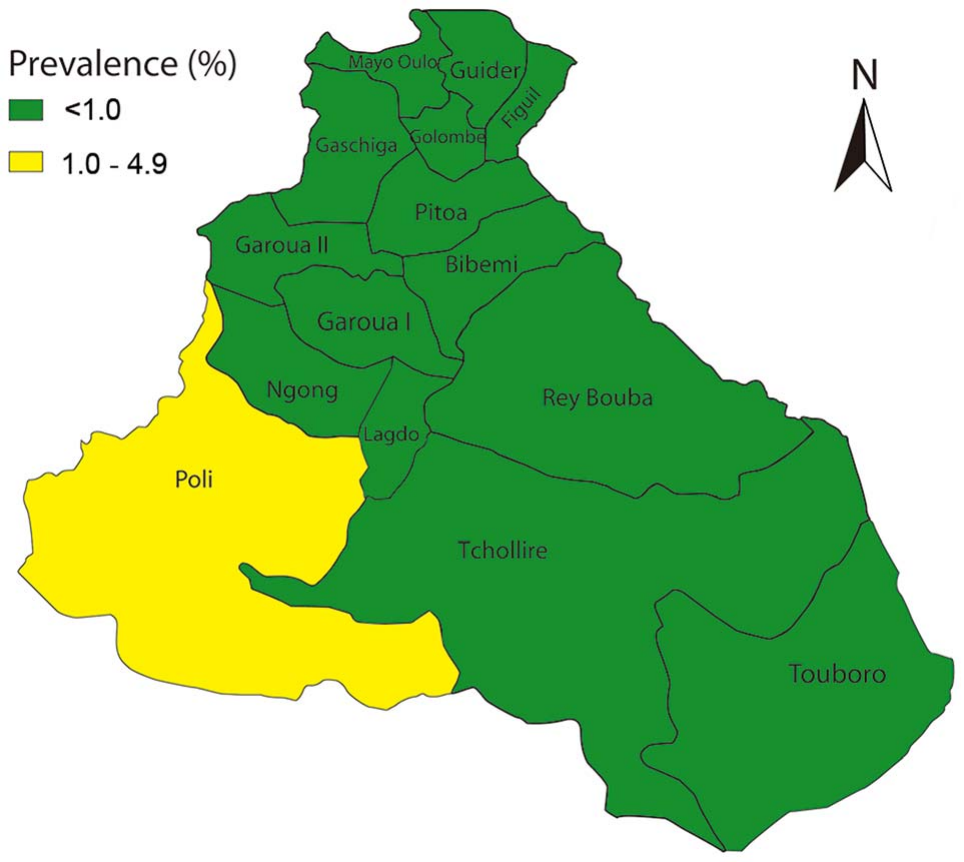
TT prevalence in the North Region Cameroon.

The prevalence of TT in people aged 15 and above was 0.25% (95% CI: 0.20–0.33%) in the region, ranging from 0% to 1.8% in 15 districts surveyed. Four (4) HDs in the region had a TT prevalence of ≥0.1%, and these were Poli, Rey Bouba, Tcholliré and Touboro HDs (Figure 2). Poli district was the only HD with a prevalence of TT >1%.

The prevalence of TT in women was 0.3% (95% CI: 0.2–0.4) and that in men was 0.2% (95% CI: 0.1–0.3%) respectively. These were not statistically significantly different (p>0.05). The prevalence of CO, low vision and blindness among individuals aged 15 and above were 0.22% (95% CI: 0.17–0.29%), 0.11% (95% CI: 0.07–0.17%), 0.01% (95% CI: 0.00–0.03%) respectively. There was no significant difference between women and men in any of these (p>0.05), however there was a trend of increasing prevalence of TT and CO with age (Table 3).

**Table 3:** Prevalence (%) of TT, CO, low vision and blindness due to trachoma in people of ≥15 years.

	TT % (95% CI)	CO % (95% CI)	Low vision % (95% CI)	Blindness % (95% CI)
***By age group***				
15–24	0.0	0.0	0 (0.00–0.05)	0.0 (0.00–0.05)
25–34	0.1 (0.04–0.23)	0.0	0 (0.00–0.08)	0.0 (0.00–0.08)
35–44	0.3 (0.17–0.55)	0.2 (0.10–0.41)	0.16 (0.07–0.37)	0.0 (0.00–0.12)
45–54	0.4 (0.21–0.71)	0.4 (0.21–0.71)	0.09 (0.02–0.33)	0.0 (0.02–0.33)
55–64	0.9 (0.55–1.46)	0.6 (0.35–1.10)	0.20 (0.07–0.58)	0.1 (0.01–0.37)
65–74	1.5 (1.00–2.33)	1.5 (1.00–2.33)	1.09 (0.63–1.90)	0.1 (0.02–0.51)
≥75	0.1 (0.02–0.32)	0.2 (0.09–0.51)	0.14 (0.04–0.50)	0.0 (0.00–0.26)
***By sex***				
Male	0.2 (0.1–0.3)	0.2 (0.1–0.2)	0.12 (0.06–0.21)	0.01 (0.00–0.06)
Female	0.3 (0.2–0.4)	0.2 (0.1–0.3)	0.11 (0.06–0.18)	0.01 (0.00–0.05)
**Total**	**0.25 (0.20–0.33)**	**0.22 (0.17–0.29)**	**0.11 (0.07–0.17)**	**0.01 (0.00–0.03)**

### Estimation of trichiasis cases in the region

Overall prevalence of TT among all ages examined was shown in Table 2, with an overall prevalence of 0.1% (95% CI: 0.1–0.2). The number of TT cases in each district was estimated using the overall TT prevalence in each district. There are estimated 1245 cases of TT in the region (Table 2). Most of the cases are concentrated in four HDs, representing 83% of the total cases in the region. These HDs are Garoua 1 (259), Poli (391), Tcholliré (217) and Touboro (187).

### Treatment needed for the North Region

On the basis of the population census data in 2010 and the growth rate of Cameroon population (2.6%), population figures for the three endemic HDs in different years were projected and the number of treatments needed for each endemic HD was calculated. In total, 957,033 treatments with Zithromax will be needed for the three endemic HDs from 2013 to 2015, including 76,563 bottles of tablets, 61,450 bottles of pediatric oral suspension and 25,183 tubes of tetracycline eye ointment (Table 4).

**Table 4:** Treatment needed for the three endemic health districts.

Health District	TF %	Number of treatments needed per year			Gross required amount per year								
					Number of tablets (bottle)			Pediatric Oral Suspension (bottle)			Tetracycline Eye Ointment		
		2013	2014	2015	2013	2014	2015	2013	2014	2015	2013	2014	2015
POLI	12.6	83189	85352	87572	6655	6828	7006	4991	5121	5254	1664	1707	1751
REY BOUBA	10.6	105716	108465	111285	8457	8677	8903	6343	6508	6677	2114	2169	2226
TCHOLLIRE	14.5	138328	141924	145614	11066	11354	11649	8300	8515	8737	2767	2838	2912
Total		327233	335741	344471	26179	26859	27558	21647	20144	22683	8558	8729	8904

## Discussion

It is critically important to conduct disease mapping surveys to gather trachoma prevalence data at district level for planning the national trachoma elimination program in order to achieve the goal of eliminating trachoma as a blinding disease by year 2020. To facilitate the national planning of Cameroon trachoma elimination program, the mapping survey of trachoma prevalence was carried out in the North Region in Cameroon. Overall participation in the survey was very high, 99% for the sample of children aged 1–9 years and 99.1% for the population aged 15 years and above. A participation rate of 85% is generally acceptable. The high participation rate observed in this survey may be due to the short time elapsed between enumeration and examination. On the other hand, the high proportion of females (55.7%) to males in the adults examined suggests that many men may have been away from home for professional reasons. Therefore, some of those who were not at home may not have been enumerated at the time of the survey team's visit.

The prevalence of TF for the 15 HDs surveyed was 4.2%, and that of TT was 0.2%, but in three HDs the prevalence of TF was ≥10%; the prevalence of TF ranges from 0% (Mayo Oulo and Golombe HDs) to 14.5% (Tcholliré HD). This is not surprising as it is known that distribution of trachoma is rather focal and the prevalence of trachoma may vary between districts and even between communities [17]. This survey has identified additional 3 HDs with prevalence of TF in children aged 1–9 years ≥10%, qualifying for full community-based implementation of the A, F and E components of SAFE strategy. A total population of 302 981 inhabitants in these HDs need to be targeted each year for mass antibiotic treatment for three years as from 2013. This is in addition to the 1.67 million in 13 HDs in the Far North region already targeted each year for treatment for at least three years [8].

It has been believed that trachoma is only endemic in the northern part of Cameroon according to clinical records and observations. This part of the country shares borders with northern Nigeria and Chad where trachoma is endemic [18], [19], [20], [21]. In northern Nigeria, the prevalence of TF among children 1–9 years was 37% in Sokoto State [19], 18.3% in Yobe State [20], and 5–24% in Katsina State [18]. In Chad, TF prevalence of 31.5% in children under 10 years of age was reported [21]. The previous surveys in the Far North region showed TF prevalence of between 0 to 42.5%, and the overall prevalence of TF found in this survey is lower than that found in the Far North region [7], [8]. It is also lower than the results from the surrounding countries, such as in Chad, Niger and Nigeria [18], [21], [22]. A separate survey in the Adamawa region revealed an overall prevalence of TF <5% (Noa Noatina et al. unpublished results), while trachoma cases are not usually reported in the seven other regions of Cameroon. This shows that trachoma is mainly distributed in the Far North and gradually becomes insignificant towards the south of the country. Rural settings, isolation, remoteness from urban areas are risk factors for the occurrence of active trachoma. Children with signs of trachoma are more likely to be found in least populated and remote villages. This is because of less socio economic development, lack of health facilities and less hygienic practice.

The total prevalence of TT in the North Region in people aged 15 years and above was 0.2%. It ranged from 0 to 1.9%. Poli district was the only HD with a prevalence of TT≥1%. This was lower than the prevalence of TT found in the Far North Region (1%) [7], [8], and in Chad where the prevalence of TT was 1.5% in women aged 14 and above [21]. In Katsina state of Nigeria the prevalence of TT in adults aged 15 years and above ranged from 2.3 to 8.0% [18]. The fact that there were 8 HDs with a TT prevalence of ≥0.1% suggests that TT is a public health problem in these districts and that implementation of “S” component of the SAFE strategy is needed. TT prevalence increases with age and the peak is reached in the 65–74 age group for both women and men. In The Gambia, older age was a significant risk factor for development of trichiasis, corneal opacity, and visual loss [23]. There seemed to be more women having TT (0.3%) compared with men (0.2%), but the difference was not statistically significant. The lack of significant difference between women and men may be attributed to the overall low TT prevalence in the region and relatively few TT cases found in the samples surveyed, as it is well known that women have a greater burden of trichiasis than men [24], [25]. From the survey data, we estimate that there are 1245 people suffering from TT in the region, who are at the risk of going blind and need surgical corrections.

The prevalence of CO (0.2%), and blindness (0.0%) in the sample of people aged 15 years and above were lower than those found in Chad, 1% and 0,5% respectively [21], or in the Far North region of Cameroon [7], [8].

The study had a number of limitations. Firstly, for the training of surveyors (trachoma graders), we used pictures showing the various forms of trachoma without having field grading exercises using trachoma patients. Grading for active trachoma can be learned from pictures, but grading in the field in population may be more difficult. On the other hand, TT may be difficult to recognize on photos as graders may need to look from the side to identify the in-turned lashes. As a result, there may have been some imprecision in the estimation of both signs. However, the graders had acquired great experience from their daily activity and previous surveys and only those who obtained at least 90% of answers in correctly grading trachoma during training sessions were finally recruited as trachoma graders in the survey team. Therefore, the overall prevalence of TF and TT may have been slightly but should not have been significantly underestimated.

Secondly, this survey did not involve environmental and socio economic risk factors for trachoma occurrence. The emphasis was to determine trachoma prevalence for planning and implementing the interventions needed to eliminate trachoma in the region. Therefore it was not possible to make a correlation between our findings and trachoma risk factors. Assessment of water and sanitation would be useful for designing implementation of “F” and “E” components of the SAFE strategy for long lasting elimination of blinding trachoma.

The mapping survey has filled the knowledge gap in trachoma distribution in the North Region. It has enabled the Ministry of Public Health to assess the magnitude and the distribution of the disease in the region to design a national plan for elimination of trachoma. A national strategic plan for trachoma control has recently been finalized. In the North Region, the four components of the SAFE strategy will be implemented in 3 HDs with TF prevalence of ≥10%: Poli, Rey Bouba, Tcholliré. Mass drug administration with azithromycin should start in 2013 in these HDs. In those HDs where the prevalence of TF is less than <5%, sub-district level survey may be needed to identify potential hot spot communities for intervention and implementation of “F” and “E” components of the SAFE strategy may be necessary. Those individual TF cases identified may be attended through existing PHC services. Effort must be made to provide surgical operations to the TT cases in all HDs to prevent them from becoming blind. With the help of Sightsavers and other partners, surgical campaigns are currently held in the Far North region to address the backlog of trichiasis in endemic HDs. And we hope the same effort will prevail in the North Region.

### Conclusion

This survey has provided baseline data for the planning and implementation of trachoma control activities in the North Region. The overall prevalence of TF is 4.2% and the overall prevalence of TT is 0.2%; three HDs had a TF prevalence of ≥10%. These three HDs were eligible for mass drug administration with azythromycin, along with the implementation of the “F” and “E” components of the SAFE strategy.

## Supporting Information

Checklist S1 **STROBE checklist.**
(DOCX)
